# F64. DISRUPTION IN WORKING MEMORY GATING OBSERVED IN SCHIZOPHRENIA

**DOI:** 10.1093/schbul/sby017.595

**Published:** 2018-04-01

**Authors:** Rachael Blackman, Noah Philip, David Badre

**Affiliations:** 1 Alpert Medical School, Brown University; 2 Brown University

## Abstract

**Background:**

Working memory and cognitive control deficits are hallmarks of schizophrenia. It is not known specifically how gating mechanisms that regulate memory may be disrupted in schizophrenia. Gating mechanisms determine what task-relevant information is selected into working memory while distractors are left out (input gating) and which items stored in working memory are selected for the rule or goal at hand (output gating). The current study investigated whether patients are able to perform the same cognitive control task that is able to dissociate input and output gating processes in a general population, and explored whether schizophrenia patients inappropriately use suboptimal cognitive control strategies (e.g. output gating when input gating can be used).

**Methods:**

Patients (n=5) with schizophrenia or schizoaffective disorder were recruited from the Providence VAMC. Participants completed a computer-based cognitive control task. In this task, participants remembered a target item from a sequence of two items in order to select a response. A context (rule) cued which item was relevant to remember, and was presented first in the stimulus sequence (context first) or last (context last). On selective trials, one item in the trial was relevant. On global trials, both items in the trial were relevant.

**Results:**

Patients were able to complete the task with minor modifications to adjust for ability to understand the task rules. Preliminary results of reaction time data suggest that patients were challenged at increased cognitive load. Patients performed poorly on trials where participants could use only an input gating strategy (selective first). Preliminary data also suggest that performance in patients tended to be slightly worse for selective first trials where the distractor was presented before the relevant item (i.e. on trials where input gating would be required to keep the distractor out of working memory).

**Discussion:**

The current study supports the feasibility of using the cognitive control task selected to investigate gating mechanisms in the schizophrenia patient population. Preliminary data suggest disruption in the ability for patients to optimally use gating strategies and handle cognitive load. Future research will seek to reproduce these preliminary results in a larger sample, as well as compare patient performance to an age-matched control population directly. By understanding how gating mechanisms are disrupted in the patient population, we may be able to better develop therapeutic interventions such as cognitive training strategies to treat cognitive dysfunction in schizophrenia patients.

